# Dr. George S. Wyckoff

**Published:** 1914-06

**Authors:** 


Dr. George S. Wyckoff, Buffalo, 1877; died in Pittsburgh, February 10, aged 63, of heart disease. He had practiced at Leechburg, Pa.

